# Viral Characteristics Associated with the Clinical Nonprogressor Phenotype Are Inherited by Viruses from a Cluster of HIV-1 Elite Controllers

**DOI:** 10.1128/mBio.02338-17

**Published:** 2018-04-10

**Authors:** Concepción Casado, Sara Marrero-Hernández, Daniel Márquez-Arce, María Pernas, Sílvia Marfil, Ferran Borràs-Grañana, Isabel Olivares, Romina Cabrera-Rodríguez, María-Soledad Valera, Laura de Armas-Rillo, Philippe Lemey, Julià Blanco, Agustín Valenzuela-Fernández, Cecilio Lopez-Galíndez

**Affiliations:** a Unidad de Virologia Molecular, Laboratorio de Referencia e Investigación en Retrovirus, Centro Nacional de Microbiología (CNM), Instituto de Salud Carlos IIII, Majadahonda, Madrid, Spain; b Laboratorio de Inmunología Celular y Viral, Unidad de Virología IUETSPC, Unidad de Farmacología, Sección de Medicina, Facultad de Ciencias de la Salud, Universidad de La Laguna (ULL), Tenerife, Spain; c Institut de Recerca de la Sida IrsiCaixa, Institut d’Investigació en Ciències de la Salut Germans Trias i Pujol (IGTP), Badalona, Spain; d Universitat de Vic, Universitat Central de Catalunya, UVIC, Vic, Spain; e Department of Microbiology and Immunology, Rega Institute, KU Leuven, University of Leuven, Leuven, Belgium; Medical School, University of Athens

**Keywords:** CD4 binding, HIV-1, heritability, LTNP-EC, actin-tubulin modifications, cell signaling, phylogenetic analysis, viral envelope

## Abstract

A small group of HIV-1-infected individuals, called long-term nonprogressors (LTNPs), and in particular a subgroup of LTNPs, elite controllers (LTNP-ECs), display permanent control of viral replication and lack of clinical progression. This control is the result of a complex interaction of host, immune, and viral factors. We identified, by phylogenetic analysis, a cluster of LTNP-ECs infected with very similar low-replication HIV-1 viruses, suggesting the contribution of common viral features to the clinical LTNP-EC phenotype. HIV-1 envelope (Env) glycoprotein mediates signaling and promotes HIV-1 fusion, entry, and infection, being a key factor of viral fitness *in vitro*, cytopathicity, and infection progression *in vivo*. Therefore, we isolated full-length *env* genes from viruses of these patients and from chronically infected control individuals. Functional characterization of the initial events of the viral infection showed that Envs from the LTNP-ECs were ineffective in the binding to CD4 and in the key triggering of actin/tubulin-cytoskeleton modifications compared to Envs from chronic patients. The viral properties of the cluster viruses result in a defective viral fusion, entry, and infection, and these properties were inherited by every virus of the cluster. Therefore, inefficient HIV-1 Env functions and signaling defects may contribute to the low viral replication capacity and transmissibility of the cluster viruses, suggesting a direct role in the LTNP-EC phenotype of these individuals. These results highlight the important role of viral characteristics in the LTNP-EC clinical phenotype. These Env viral properties were common to all the cluster viruses and thus support the heritability of the viral characteristics.

## IMPORTANCE

HIV-1 long-term nonprogressor elite controller patients, due to their permanent control of viral replication, have been the object of numerous studies to identify the factors responsible for this clinical phenotype. In this work, we analyzed the viral characteristics of the envelopes of viruses from a phylogenetic cluster of LTNP-EC patients. These envelopes showed ineffective binding to CD4 and the subsequent signaling activity to modify actin/tubulin cytoskeletons, which result in low fusion and deficient entry and infection capacities. These Env viral characteristics could explain the nonprogressor clinical phenotype of these patients. In addition, these inefficient *env* viral properties were present in all viruses of the cluster, supporting the heritability of the viral phenotype.

## INTRODUCTION

Natural HIV-1 infection shows a wide range of disease outcomes that result in the classification of patients according to progression time. A small group of HIV-1-infected individuals, called long-term nonprogressors (LTNPs), and especially a subgroup of LTNP elite controllers (LTNP-ECs), display permanent control of viral replication and lack of clinical progression. This control is the result of a complex interaction of host, immune, and viral factors.

In HIV-1 patient classifications, LTNPs are individuals infected with HIV-1 for more than 10 years, maintaining high CD4^+^ lymphocyte numbers without clinical symptoms, and remaining therapy naive (1). Within the LTNP group and according to HIV-1 plasma viral load, we can distinguish LTNP noncontrollers (LTNP-NCs) with viral loads above 2,000 copies/ml, LTNP viremic controllers (LTNP-VCs) with viral loads between 50 and 2,000 copies/ml, and LTNP-ECs with undetectable viral loads (<50 copies/ml) (2). The latter definition has been used for the selection of the individuals of this study. Patients with very similar characteristics are also called elite suppressors (3) or HIV-1 controllers (HICs) (4). Progressor patients are individuals with a symptomatic infection or with the initiation of antiretroviral therapy (ART) within 10 years after seroconversion and a minimum of 3 determinations above 2,000 copies/ml (2). Different nomenclatures have been used to name HIV-1 patients by distinct groups, and these definitions are reviewed in reference 5.

The LTNP phenotype has been associated with host genetic background, principally HLA-B genotypes—mostly HLA-B57/B58 or -B27 (6) and HLA-C (2). Immunologic studies demonstrated potent and broad cytotoxic T lymphocyte (CTL) responses in LTNPs (7). In addition, viral factors were also identified in groups of LTNPs, like viruses with important deletions in the *nef* gene in a cohort of Australian nonprogressors (8) or with mutations in different genes (9). In general, viruses with low replication capacity are detected in LTNPs (10–13).

Plasma viral load in infected individuals was found to be a good predictor of progression to AIDS (14), and the set point viral load (SPVL), or the stable quantity of the virus in the patient’s blood after primary infection, has been directly correlated with infectiousness and virulence (15). SPVL in HIV-1 patients ranges over several orders of magnitude and is a key determinant of disease progression. A number of recent studies reported the high heritability of the SPVL, implying that viral genetic factors contribute substantially to the overall variation in viral load. From a virological point of view, “heritability” is the fraction of variability in disease outcome explained by pathogen genetics, because these factors are “inherited” by the new host upon infection (16). In transmission pair studies, viral properties and fitness were inherited in the recipients, as shown by the similarity of viral loads in linked individuals (17–20). Viral heritability has also been inferred by phylogenetic approaches (17), but discordant values were obtained in different studies from distinct locations ranging from 6 to 59% (16), raising the question of how effectively HIV-1 phylogenies can be used to measure heritability (21). A modeling approach by Bonhoeffer et al. demonstrated that high heritability is the most parsimonious explanation for the observed variance of SPVL (22). In a standardized study with 2,028 samples from different European countries, viral genetic variation accounts for a third of variability in HIV-1 SPVL in Europe (16). Most of the research on heritability in HIV-1 has either focused on transmission pairs or on phylogenetic signal in large cohorts of patients. Although it could bridge the gap between these two extremes, heritability in HIV-1 transmission clusters has to our knowledge not been demonstrated so far.

It has been reported that HIV-1 envelope glycoprotein (Env) functions, such as tropism or fusogenicity are linked to *in vitro* HIV-1 cytopathicity (23, 24) and viral fitness (25, 26), as well as to clinical progression of HIV-1 infection (27) and simian immunodeficiency virus (SIV) infection *in vivo* (28). Moreover, we and other groups have previously reported that HIV-1 Env-gp120-induced posttransduction modifications and reorganization of tubulin and actin cortical cytoskeleton are key signals and processes to promote efficient early HIV-1 infection (29–34). These Env/CD4-mediated events increase the probability of HIV-1 Env-CD4/coreceptor interactions, potentiating fusion pore formation, and thus HIV-1 entry and infection. Therefore, it is plausible to propose that HIV-1 Env-mediated early functions could be responsible for an inefficient HIV-1 infection and viral progression *in vivo*, being determinant for the LTNP-EC clinical phenotype in some HIV-1^+^ individuals.

**FIG 1:**
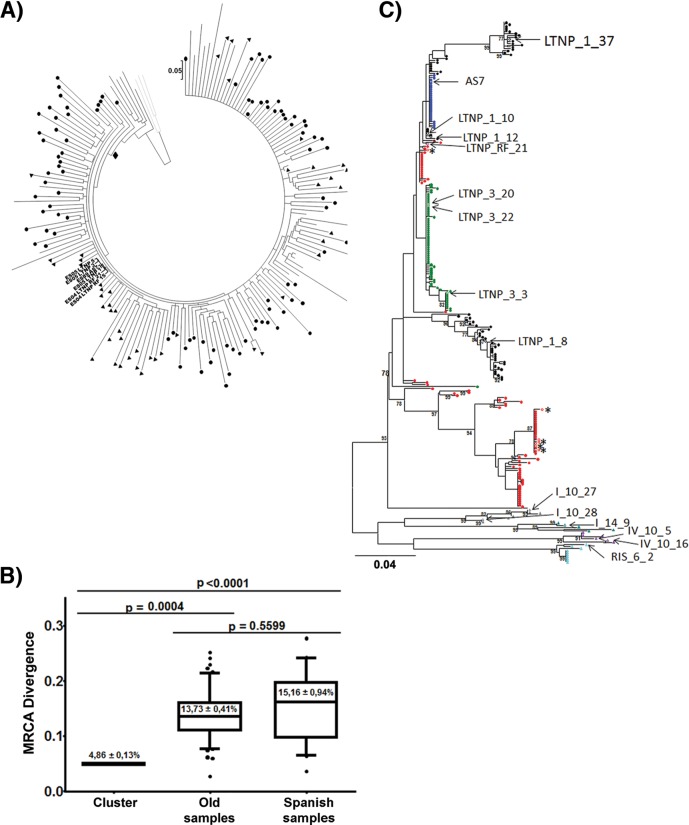
Phylogenetic and genetic distance analyses of the sequences from the cluster viruses to the MRCA. (A) Phylogenetic tree with complete *env* sequences from the Bayesian Markov chain Monte Carlo (MCMC) analysis (MrBayes). The 50% majority rule consensus obtained by Bayesian MCMC analysis is shown, and the most recent common ancestor (MRCA), including the vast of majority sequences analyzed, is marked (solid diamond). Branch lengths represent the mean value observed for that branch among the postburning sampled trees. The symbols identify nucleotide sequences analyzed: solid circles correspond to old sequences collected before 1995, and solid triangles correspond to Spanish sequences collected from 1989 to 2005. Only the Spanish cluster sequences collected between 2004 and 2005 are identified by name. Gray lines are subtype D sequences used as an outgroup. (B) Genetic distance of the sequences to the MRCA. MRCA genetic distances of the cluster LTNP-EC viruses studied were compared with the MRCA genetic distances of viruses obtained from HIV-1-infected individuals before 1995 (old samples) or from Spanish HIV-1-infected individuals taken from 1989 to 2005 (35). Genetic distance was calculated as the MRCA-to-tip distances from the MrBayes phylogenetic tree using TreeStat v.1.2 (http://tree.bio.ed.ac.uk/software/treestat/). Boxes represent the 25 to 75% of the mean, and the whiskers indicate 5 and 95%. The panel shows *P* values for nonparametric Dunn’s test for multiple comparisons. (C) Maximum likelihood phylogenetic trees of patients’ viral sequences in the *env* gene. Shown are partial HIV-1 PBMC-derived gp120 *env* sequences of cluster patients LTNP_1 (black circles), LTNP_3 (green circles), LTNP_5 (red circles), LTNP_RF_15 (brown circles), LTNP_RF_21 (pink circles), and AS7 (blue circles) and progressor patients I_10 (gray triangles), I_14 (teal triangles), IV_10 (purple triangles), and RIS_06 (turquoise triangles). The clones analyzed are indicated by open symbols and arrows. Asterisks indicate nonfunctional clones. Numbers at branch nodes refer to the bootstrap support. (Only values greater than 70% are shown.) Branch lengths are drawn to scale.

In a previous study, we identified within a large group of Spanish HIV-1 LTNP patients a cluster of 5 patients with an LTNP-EC clinical phenotype (see Fig. 1A). These individuals were infected for more than 25 years with very similar viruses, all showing low replication characteristics and 11 unusual amino acid changes in the envelope protein of the viruses and linked by clinical characteristics, risk practices, and epidemiological characteristics, identifying a transmission cluster (35).

For these reasons, in this work, we performed a comprehensive functional characterization of Envs from these LTNP-EC viruses, focusing on the initial events of viral infection: binding of Env to CD4^+^ cells, Env-triggered early signals for actin and α-tubulin cytoskeleton modulation, and the subsequent membrane fusion and early infection in target cells. We observed that the genotypic and phenotypic characteristics of the Env proteins from the HIV-1 viruses of the LTNP-EC cluster were inherited by all the patients’ viruses and produced, in individuals with different host genotypes, the same clinical LTNP-EC phenotype.

## RESULTS

### Low replication and transmission of the viruses from the LTNP cluster.

In a previous study, we described a cluster of 6 HIV-1^+^ individuals, 5 of them with known LTNP characteristics, using a Bayesian phylogenetic reconstruction from complete *env* gene sequences; this cluster was supported by a posterior probability of 0.87, and it increased to 0.98 when patient LTNP_RF_15 was not included (35) (Fig. 1A). The mean genetic distance among the cluster viruses was 0.72%, and the mean genetic distance to the cluster’s most recent common ancestor (MRCA) was 0.96%.

The *env* gene nucleotide sequences from these patients (35) displayed very short branches in the phylogenetic tree, indicating limited viral replication, in comparison with other LTNP patients from Spain or old viruses from Europe and North America (Fig. 1A). When we calculated the mean divergence of all the *env* gene sequences studied from the subtype B most recent common ancestor (MRCA) of the tree, samples from the cluster individuals, although obtained in 2004 and 2005, showed a divergence of 4.86% substitutions per nucleotide (S/N) (Fig. 1B). In contrast, old chronic progressor HIV-1 individuals, sampled before 1995, displayed a divergence of 13.73% S/N, and Spanish samples, obtained between 1998 and 2005, showed a divergence of 15.16% S/N (Fig. 1B). Genetic distance from each nucleotide sequence was calculated as the MRCA-to-tip distances from a MrBayes phylogenetic tree using TreeStat v.1.2 (35). Because of the low fidelity of the reverse transcriptase, HIV-1 replication is inevitably linked to the generation of variability (36). In phylogenetic reconstructions, the accumulation of variability results in a progressive increase of the genetic divergence from MRCA, visualized by the branch length (37–39). The very short branch length and the limited divergence of the sequences from the cluster viruses (Fig. 1B) led us to conclude that these viruses went through a comparatively very limited number of replication cycles. This result is even more relevant considering that samples were taken at least 15 years after primary infection (Table 1) and pointed to a potential functional defect in the viruses from the LTNP HIV-1 individuals that could impair replication capacity and transmissibility.

**TABLE 1:** Epidemiological, clinical, and host characteristics of the patients

Patient	Sex	Origin	Hospital	Transmission^a^	Yr first HIV-1^+^	Sample date	Group	Viral load (copies/ml)	CD4^+^ count (T cells/µl)	HLA-B genotype
LTNP_1	F	Madrid	C. S. Sandoval	IDU	1990	2005	LTNP-VCs	125	628	B1501/B2705
LTNP_3	M	Madrid	C. S. Sandoval	IDU	1988	2005	LTNP-ECs	<50	880	B2705/B5801
LTNP_5	M	Madrid	C. S. Sandoval	IDU	1986	2005	LTNP-ECs	<50	559	B2705/B3503
LTNP_RF_15	M	Madrid	12 de Octubre	IDU	1989	2004	LTNP-ECs	<50	460	B44/B57
LTNP_RF_21	M	Madrid	12 de Octubre	IDU	1985	2004	LTNP-ECs	<50	690	B47/B14
AS7	F	NK^b^	El Patriarca	IDU	1989	1989	NK	NK	NK	B3501/B5101
I_10	F	Madrid	C. S. Sandoval	IDU/HT	NK	1993	Progressors	8.9 × 10^4^	251	
IV_10	M	Vigo		IDU	1991	1994	Progressors	1.7 × 10^6^	342	
I_14	F	Madrid	C. S. Sandoval	IDU/HT	1987	1994	Progressors	1.3 × 10^5^	337	
RIS_06	M	Madrid	C. S. Sandoval	MSM	2005	2005	Progressors	7.6 × 10^4^	436	

a Transmission route: IDU, intravenous drug user; MSM, men who have sex with men; HT, heterosexual.

b NK, not known.

### Functional analysis of Env from HIV-1 viruses from the LTNP-EC cluster.

To investigate the causes of the limited viral replication and the restricted *env* sequence evolution in the cluster HIV-1 viruses, we studied the phenotypic characteristics of viral Env from the patients. The nucleotide sequences of three of the cluster patients (LTNP_1, LTNP_3, and LTNP_5) have been extensively investigated in the laboratory during a follow-up of at least 7 years (13, 40). In these studies, we detected a limited evolution of the sequences, but different viral subpopulations were identified in the tree, particularly in patients LTNP_1 and LTNP_5 (Fig. 1C). For the functional analysis of the viral Envs, we selected 4 clones in patient LTNP_1 (Fig. 1C) and 3 clones in patient LTNP_3 (Fig. 1C) corresponding to the different lineages found in these patients. Patient LTNP_5 also showed a tree with distinct lineages but all the recovered clones were nonfunctional (Fig. 1C, asterisks). For patient AS7, the sole patient without clinical data and follow-up, we only obtained a sample in 1989 and given the low diversity found in the quasispecies of this sample, a single clone was selected (Fig. 1C). For patients LTNP_RF_15 and LTNP_RF_21, we only had one clone from each patient.

The clones from the progressor patients were obtained from Spanish HIV-1 patients without follow-up, and we have no information on the heterogeneity of the quasispecies, although the trees showed long tip branches. The clones analyzed from each patient are marked in Fig. 1C with arrows, and when possible, we included at least two clones from each control patient.

**FIG 2:**
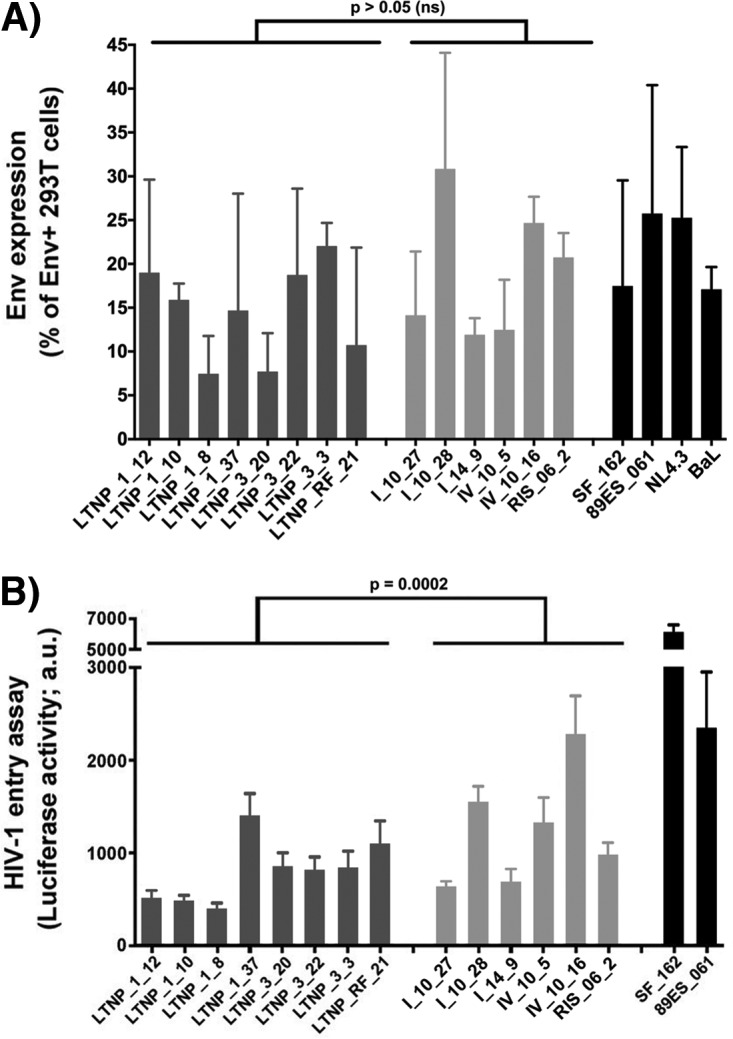
Analysis of the expression and viral entry efficiency of the different HIV-1 Env proteins from LTNP-EC and control patients. (A) Flow cytometry analysis of the cell-surface expression level of the assayed HIV-1 Envs in 293T cells. (B) Luciferase-based assay of viral entry and infection in permissive CEM.NKR-CCR5 cells by nonreplicative HIV-1 luciferase reporter pseudoviruses bearing viral Envs from cluster LTNP-ECs (dark gray bars) or progressors (light gray bars). SF162 and 89ES_061 are reference strains (black bars). All data were corrected by the nonproductive infection values (baseline) obtained with a neutralizing anti-CD4 MAb (5 μg/ml), under the same experimental conditions. Data are values from 10 independent experiments carried out in triplicate and comparing median values between groups (nonparametric Mann-Whitney test). a.u., arbitrary light units.

To determine whether the Envs, isolated from HIV-1 viral particles of different individuals, are responsible for the poor ability of these viruses to replicate, we first expressed Envs isolated from primary LTNP-EC and chronic progressor viruses in 293T cells, in order to analyze their cell surface expression. Transfected cells stained with monoclonal antibody (MAb) 2G12 showed a median value of the Env expression from the LTNP-EC viruses not statistically different from the chronic progressor viruses and comparable to reference Envs (Fig. 2A). Therefore, the inefficacy of HIV-1 viruses from LTNP-EC individuals to replicate cannot be attributed to a difference in the Env expression level at the cell surface of cells producing virions. To further investigate this, HIV-1 luciferase-reporter pseudoviruses were produced using a pNL4.3-Luc-R-E vector, and each of the different *env* expression clones: 8 from 3 of the cluster viruses, 6 from 4 chronic progressors, and 2 from reference strains SF_162 (R5-tropic strain) and 89ES_061 (X4-tropic strain). All the *env* nucleotide sequences from the LTNP-EC cluster HIV-1 viruses showed an R5 tropism (Web PSSM; https://indra.mullins.microbiol.washington.edu/webpssm). This technical approach, using the same viral backbone with different *env* genes, allowed the generation of viral particles to infect the same permissive cells for the analysis of the functional involvement of each viral Env in infection. Equal amounts of luciferase reporter pseudoviruses were used to infect CEM.NKR-CCR5 cells, providing a quantification of HIV-1 entry in the absence of other viral factors and of any influence of late steps of viral infection (29, 30, 41). We observed that, in comparison with control and chronic progressor-derived viruses, LTNP-EC cluster-derived pseudoviruses poorly infected cells (*P* = 0.0002) (Fig. 2B). Thus, the Envs from these LTNP-EC HIV-1 viruses were unable to promote efficiently the early steps of viral infection.

**FIG 3:**
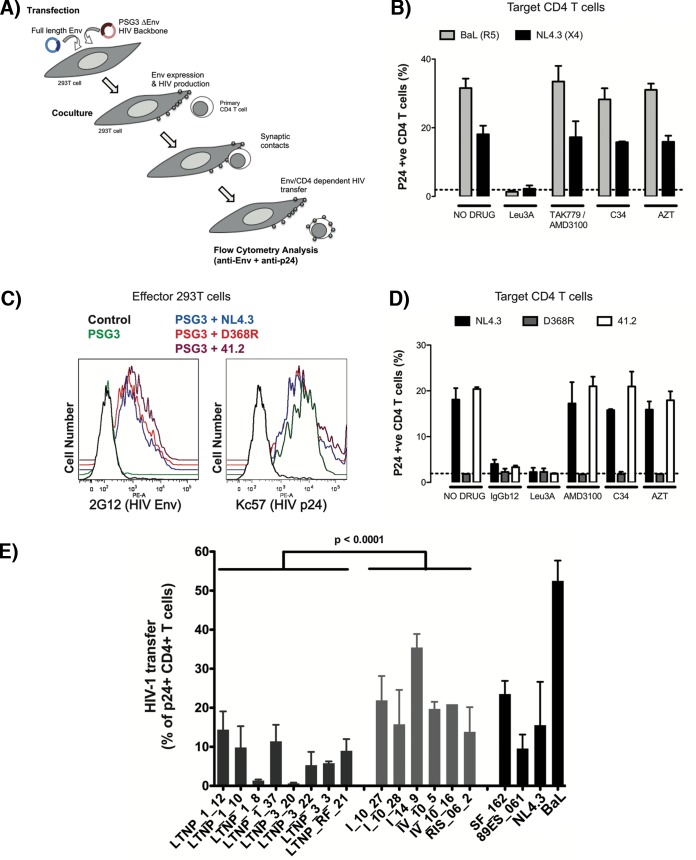
Analysis of HIV-1 Env-mediated cell-to-cell viral transfer. (A) An outline of the experimental model used for the analysis of Env-mediated cell-to-cell fusion and viral transfer is shown. Briefly, 293T cells were cotransfected with *env* and a Δ*env* HIV-1 expression plasmid. After 24 h, effector cells producing HIV-1 particles were cocultured with primary CD4^+^ T cells to force synapsis formation and massive binding of budding particles to target cells. Env expression and HIV-1 binding were analyzed by flow cytometry using anti-Env and anti-p24 antibodies, respectively. (B) HIV-1 transfer to primary CD4^+^ T cells induced by the CCR5-using Env from BaL.01 or the CXCR4-using Env from NL4.3. Cocultures were performed in the presence of the following inhibitors: 1 μg/ml of the anti-CD4 antibody Leu3A or 10 μg/ml of the CCR5 antagonist TAK779 or the CXCR4 antagonist AMD3100, the fusion inhibitor peptide C34, and the reverse transcriptase inhibitor AZT. The dotted line corresponds to background levels of signal obtained in cocultures of CD4^+^ T cells and 293T cells transfected with the Δ*env* plasmid PSG3. Data are means ± standard deviations (SD) from two different experiments. (C) Expression of HIV-1 Env in cells untransfected (control [black line]), transfected with the Δ*env* plasmid (PSG3 [green line]), or cotransfected with this plasmid and the indicated *env* from NL4.3 (blue line), the D368R mutant (red line), or the 41.2 mutant (purple line). (D) HIV-1 transfer (bound virus) to CD4^+^ T cells assessed by flow cytometry after coculture with 293T cells expressing Env from NL4.3 (black bars), the D368R mutant (gray bars), or the 41.2 mutant (open bars) and CD4^+^ T cells. Cocultures were performed in the presence of inhibitors as in panel B; the anti-gp120 antibody that blocks the CD4 binding site IgGb12 was used at 10 μg/ml. The dotted line corresponds to background levels of signal obtained in cocultures of CD4^+^ T cells and 293T cells transfected with the Δ*env* plasmid PSG3. Data are means ± SD from three different experiments. (E) Analysis of the ability to induce cell-to-cell virus transfer of HIV-1 Env proteins obtained from cluster LTNP-ECs (dark gray bars) or progressors (light gray bars) or reference strains (SF162, NL4.3, BaL.01, and 89ES_061 [black bars]). Data are from three independent experiments, comparing mean values between groups. (*P* values compare medians between groups using a nonparametric Mann-Whitney test.)

Having ruled out a defect in Env expression, we further evaluated the biological functions of these cell-surface-expressed Envs in promoting cell-to-cell HIV-1 viral transfer and infection. This process mainly relies in the first HIV-1 Env-CD4 interaction and signaling, which are key for pore fusion formation and productive viral infection (29, 30, 32, 33, 42, 43). To analyze the interaction with CD4, we first employed a viral transfer assay using unstimulated primary CD4^+^ T cells as target cells (Materials and Methods) (Fig. 3A).

In this assay, we forced the formation of virological synapses between Env- and Gag-expressing 293T cells and freshly purified primary CD4^+^ T cells. Hence, synapse formation allows budding viruses to rapidly attach to CD4^+^ T cells by a mechanism that is strictly dependent on the binding of gp120 expressed on 293T cells to the CD4 molecule on target cells (Fig. 3A). The subsequent transfer of budding particles from 293T cells to CD4^+^ T cells is independent of the coreceptor interaction or the gp41 activation (43). To fully characterize our experimental model, we used the reference Envs BaL (CCR5 tropism) and NL4.3 (CXCR4 tropism). Pharmacological inhibition with molecules that block fusion at different stages of Env function showed a complete blockade of HIV-1 binding when interfering with the gp120-CD4 interaction (with the anti-CD4 antibody Leu3A) and no effect by coreceptor fusion or postfusion blockade (CCR5 or CXCR4 antagonists TAK779 or AMD3100, gp41 inhibitor C34, or reverse transcription inhibitor zidovudine [AZT]) (Fig. 3B). Further, we used different available NL4.3 mutant derivatives that abrogate either CD4 binding capacity (the D368R mutation in gp120 [44]) or fusion capacity (the 41.2 mutation in gp41 [24]). These mutations do not alter Env expression in transfected cells (Fig. 3C). Consistent with pharmacological data, the D368R mutant completely blocked viral binding to CD4^+^ T cells, while the 41.2 mutant had no significant effect (Fig. 3D).

These data demonstrated that the assay can assess the Env-CD4 interaction, and it was therefore employed to quantify the ability of mediating CD4 binding for reference as well as Envs isolated from the LTNP-ECs and chronic progressors (Fig. 3B and E). The Envs from the HIV-1^+^ LTNP-ECs displayed a significantly lower ability (*P* < 0.0001) to transfer viral particles to primary CD4^+^ T cells than Envs from chronic progressor HIV-1^+^ individuals (Fig. 3E), suggesting that the Envs from LTNP-EC viruses had impaired binding to the cell-surface-expressed CD4 molecule.

**FIG 4:**
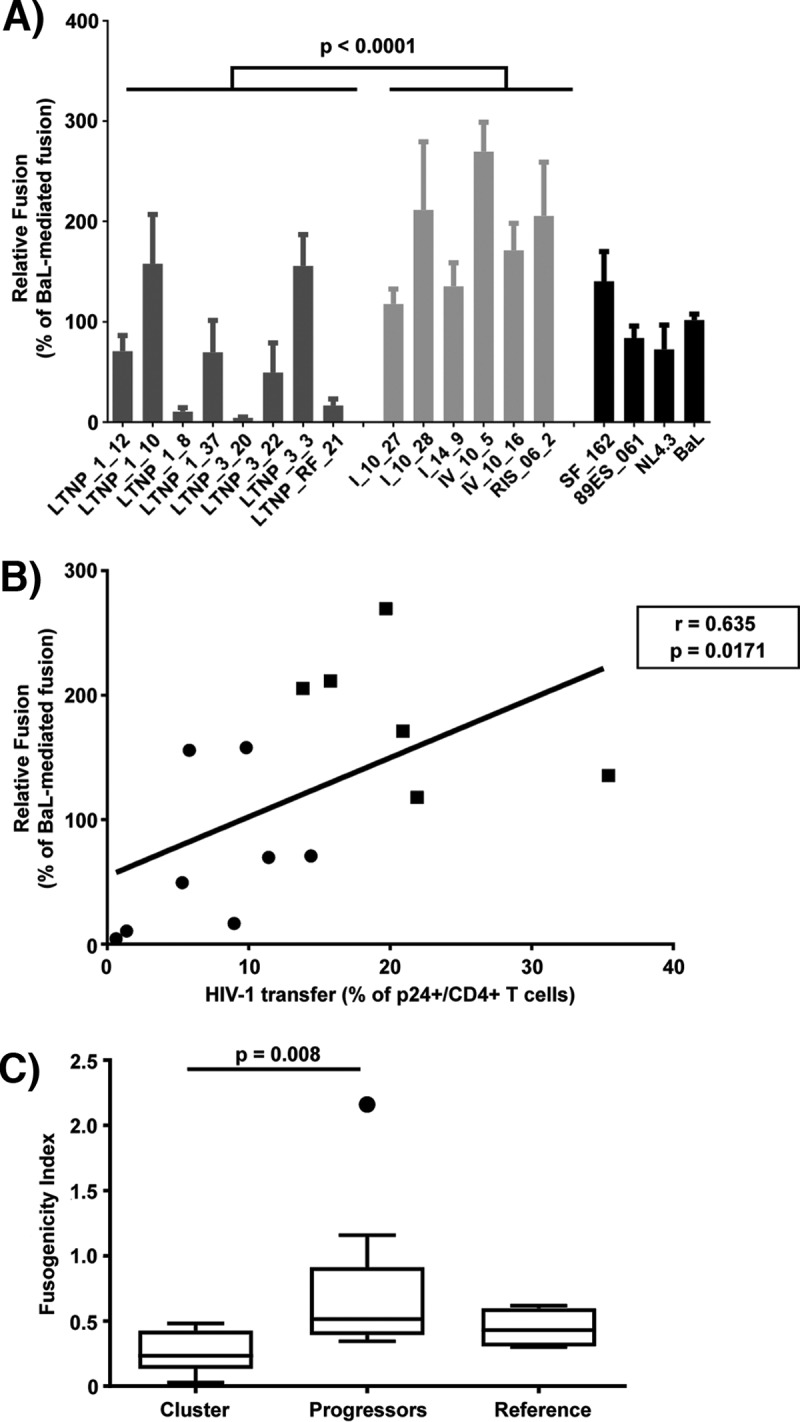
Analysis of HIV-1 Env fusogenic activity. (A) Analysis of the ability to induce cell-to-cell fusion of HIV-1 Env proteins obtained from cluster LTNP-EC (dark gray bars), progressor individuals (light gray bars), or reference HIV-1 viral strains (SF162, NL4.3, BaL.01, and 89ES_061 [black bars]). Data are from three independent experiments, comparing mean values between groups (nonparametric Mann-Whitney test). (B) Spearman’s nonparametric correlation between HIV-1 transfer activity after cell-to-cell contact (a surrogate marker of binding of the Env protein to the CD4 molecule) and the fusion capacity of the Envs obtained from the LTNP-EC cluster viruses are shown. Mean values of the three independent experiments for each Env were used for the correlation analysis. (C) A fusogenicity index (FI) was calculated for each Env clone as the ratio of fusion activity and relative fluorescence intensity. Values for LTNPs, progressors, and reference Env clones are shown: *n =* 8, 6, and 4, respectively. Boxes represent medians and interquartile ranges of values.

In addition, the fusion capacity of the same *env* clones was analyzed in cocultures between 293T transfected cells and TZM-bl cells. Consistent with pseudovirus infectivity (Fig. 2B), this assay yielded significantly lower fusion values for Envs from LTNP-EC HIV-1 viruses compared to those from chronic progressors’ HIV-1 viruses (*P* < 0.0001) (Fig. 4A). Thus, the phenotypic characterization of the Envs from the LTNP-EC cluster confirmed that the low replication capacity detected for the HIV-1 viruses in the cluster was associated with impaired Env function, thereby showing a significant lower fusogenic capacity than viruses in chronic progressors (Fig. 4A). In addition, a significant correlation was observed between the HIV-1 transfer data, mediated by Env/CD4 binding capacity and fusogenicity (*r* = 0.635, *P* = 0.0171, Spearman’s test) (Fig. 4B), thus linking the fusion defect to a low CD4 affinity. Furthermore, a fusogenicity index (FI) was calculated for each Env clone. The FI normalizes fusion values to Env expression and showed lower values in Env clones isolated from the LTNP cluster compared to progressors (Fig. 4C). Taken together, these data further confirm the deficient Env fusion capacity observed in the cluster LTNP envelopes.

### Signaling activity of Envs from the cluster HIV-1 viruses.

**FIG 5:**
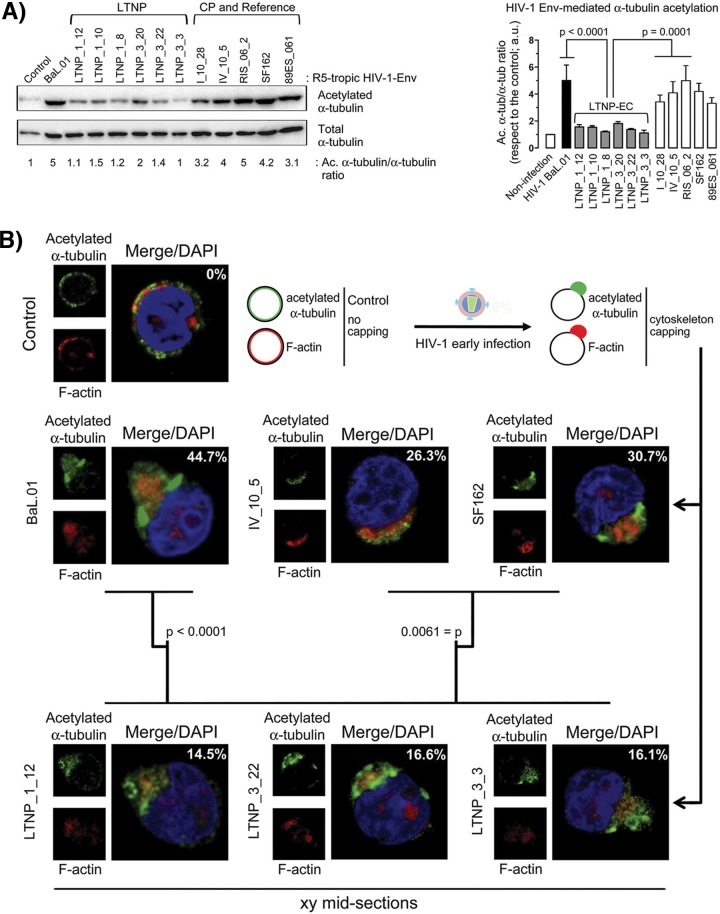
Effect of HIV-1 Env on α-tubulin acetylation and F-actin and acetylated α-tubulin reorganization, capping, and pseudopodium formation. (A, left panel) Quantitative Western blot analysis of LTNP-EC HIV-1 Env-induced α-tubulin acetylation in permissive cells compared with Envs from reference and chronic progressor (CP) patients’ viral strains, represented as acetylated α-tubulin in reference to total α-tubulin (Ac. α-tub/α-tub ratios). A representative experiment of six performed is shown. (Right panel) Histograms show the quantification of HIV-1 Env-mediated α-tubulin acetylation in permissive cells from six independent experiments carried out in triplicate. Data are means ± standard errors of the means (SEM). Acetylated α-tubulin/α-tubulin ratios are given in arbitrary light units (a.u.). (B) A series of fluorescent confocal images shows HIV-1 Env-induced redistribution of acetylated α-tubulin and actin cytoskeleton and pseudopodium formation in control, untreated, or HIV-1 Env early infected permissive cells. Merged images are shown with DAPI (4′,6-diamidino-2-phenylindole) labeling of the nucleus. A representative experiment of six performed is shown indicating the percentage of cells affected per 150 cells counted under any experimental condition. *P* values from Student’s *t* test are indicated. A scheme is presented showing the different cell morphologies and cytoskeleton profiles observed.

We next compared the signaling ability of Envs from LTNP-EC and chronic progressor HIV-1 viruses in permissive CEM.NKR-CCR5 cells, during the early virus-cell contacts in infection, by measuring the stabilization of acetylated α-tubulin. Consistent with the significantly impaired HIV-1 entry and infection promoted by LTNP-ECs’ Envs and their low CD4 binding (Fig. 2B and 3E, respectively), the Envs from LTNP-ECs promoted acetylation of α-tubulin to a smaller extent than the Env from the BaL.01 viral strain (Fig. 5A). On the contrary, viruses bearing chronic progressors’ Envs triggered acetylation of α-tubulin as efficiently as the BaL.01 Env and with a statistically higher activity than Envs from LTNP-EC HIV-1 viruses (Fig. 5A). Moreover, it has been described that efficient HIV-1 infection is achieved only when Env-triggered signals are strong enough to induce pseudopod formation by promoting actin cytoskeleton reorganization and capping in at least 20 to 30% of the cells (29, 30, 33). In an experimental model analyzed by fluorescence confocal microscopy, we observed that reference BaL.01 and SF162 Envs, as well as the chronic progressor Env from patient IV_10_5, induced acetylation of α-tubulin and F-actin capping and pseudopod formation in 44.7, 30.7, and 26.3% of cells, respectively (Fig. 5B). In contrast, representative Envs from LTNP-EC patient viruses showed an impaired ability to trigger F-actin reorganization, thereby inducing low pseudopod formation with capped reorganized actin and acetylated α-tubulin cytoskeletons in only 14 to 16% of the cells (Fig. 5B). This difference was statistically significant (*P* = 0.0061).

Analysis of the link between the low CD4 affinity and the poor fusion activity confirms that the Env from viruses of LTNP-EC patients hardly promoted the acetylation of α-tubulin compared to HIV-1 BaL.01 Env or R5-tropic Envs from chronic progressor viruses (Fig. 5A). The low stabilization of acetylated α-tubulin and the inability to reorganize F-actin and form pseudopods are consistent with the low CD4 binding, resulting in limited viral fusion and early infection (29, 30, 33, 34, 41). Similarly, R5-tropic Envs from LTNP-EC individuals promoted weak cytoskeleton posttransduction modification and reorganization and subsequent poor pseudopod formation (Fig. 5B). Considering that HIV-1 should overcome the cortical actin barrier during the early infection steps, the accomplishment of this process will predict the susceptibility of CD4^+^ T cells to infection (32). Altogether, the poor signaling activity of these Envs is consistent with their low CD4 binding and the limited Env-mediated membrane fusion and early infection described for the Envs from the LTNP-ECs.

### Analysis of the mutations responsible for the viral replicative capacity of a replicating virus from the cluster.

**FIG 6:**
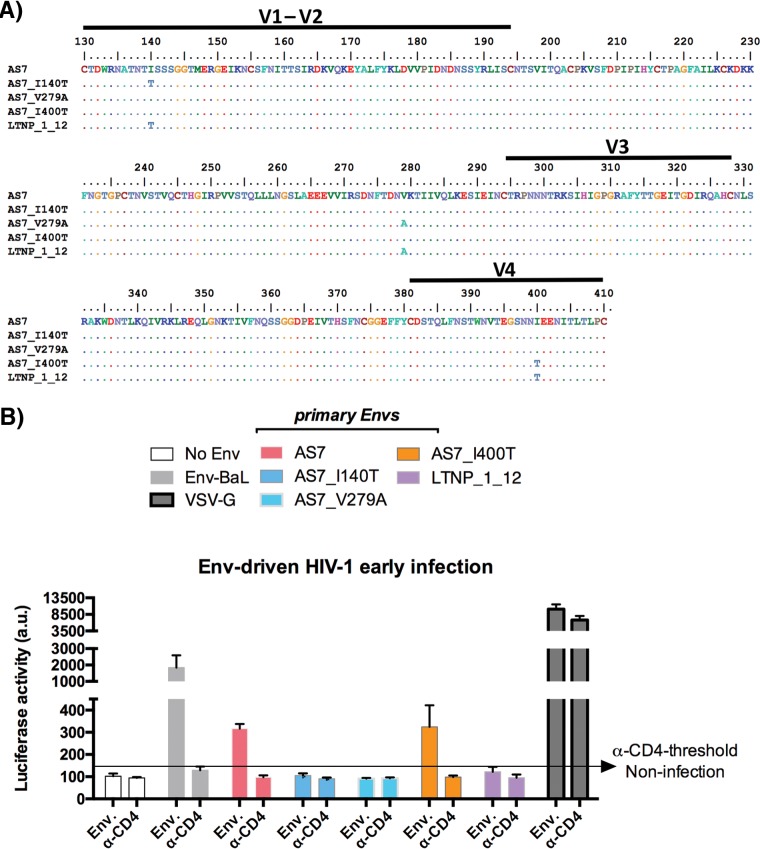
Analysis of the expression and viral entry efficiency of the different HIV-1-Env proteins from LTNP-EC and control patients. (A) Gp120 partial amino acid sequences of AS7 virus and the mutants derived in this AS7 virus backbone (AS7_I140T, AS7_V279A, and AS7_I400T) are shown and compared to the LTNP_1 clone 12 gp120 amino acid sequence from one of the cluster viruses. (B) Luciferase-based assay of viral entry and infection in permissive CEM.NKR-CCR5 cells by nonreplicative HIV-1 luciferase reporter pseudoviruses bearing viral AS7 Env and the AS7 mutants shown in panel A and the control BaL.01 Env strain (light gray bars). Nonproductive infection values (baseline) are obtained with a neutralizing anti-CD4 MAb (5 μg/ml), under the same experimental conditions. The quality of viral production and the specificity of CD4 for neutralizing HIV-1 early infection are indicated by the data obtained by using HIV-1 pseudovirus bearing the VSV-G Env. Data are values from 6 independent experiments carried out in triplicate. a.u., arbitrary light units.

To further characterize the impaired functionality of the Env from this LTNP cluster, we studied a functional Env isolated from a virus (AS7) of this cluster. Virus AS7 was derived from a cluster patient showing detectable p24 antigenemia at the sampling time (1989) and for which we do not have the clinical phenotype due to the lack of follow-up. The AS7 *env* shared 11 unusual mutations with the other *env* genes from the cluster (35), but it also displayed 3 extra mutations in the V1, C2, and V4 *env* regions. These mutations differentiated the AS7 *env* from the cluster *env* genes, as shown for the LTNP_1_12 clone (Fig. 6A). We generated in the AS7 *env* backbone three mutant viruses reverting the AS7 residue at positions 140 (V1, AS7_I140T), 279 (C2, AS7_V279A), and 400 (V4, AS7_I400T) to the residues present in the LTNP_1_12 clone (and in the other cluster viruses). The Envs with the reverting I140T and V279A mutations showed an infectious activity below the anti-CD4 threshold and a nearly defective infection (Fig. 6B), whereas the reversing I400T mutation showed a limited but detectable infection activity (Fig. 6B). These results support the inefficient signaling and functionality of the *env* genes of this cluster and their inefficient infectivity.

## DISCUSSION

LTNP-ECs are a minor group of patients who, despite being infected for many years, show natural control of HIV-1 viral replication and lack of clinical progression. Investigation of the host and viral factors and the mechanisms involved in this control is important for understanding HIV-1 pathogenesis. In this work, we analyzed the contribution of the Env complex to control viral replication and we show that viruses recovered from a cluster of LTNP-ECs displayed HIV-1 Envs with low CD4 binding, low signaling, and therefore low fusion and infection activities.

In HIV-1-infected individuals, viral fitness is a major determinant of the clinical outcome (18). Among viral proteins that determine fitness *in vitro* but also *in vivo*, the Env glycoprotein plays a major role (25), particularly in the phenotypic characteristics associated with *in vivo* depletion of CD4^+^ T cells. The Env owes its key role in viral replication to the biological activity during viral entry into target cells, which in turn determines viral tropism and cytopathic effects. The function of HIV-1 Env is a complex, multistage, and highly regulated process with many cellular molecules mobilized by the Env viral binding to CD4, CXCR4, or CCR5 receptors. These interactions result in the formation of a fusion pore through which the viral capsid enters the cell (32). The efficient pore fusion formation relies on key signals triggered by Env-CD4 interaction promoting cytoskeleton modifications (29, 30, 32, 33), such as α-tubulin acetylation (33), F-actin severing, and capping reorganization (29, 30). A deficiency in these HIV-1-Env-mediated signals leads to a defect in the early steps of viral infection and replication (29–32, 45).

Pseudoviruses bearing Envs from this cluster of LTNP-EC patients were inefficient in performing the initial steps of HIV-1 infection, pointing at a potential mechanism to explain the clinical phenotype. In fact, low viral fusion, infection, and replication activities were not the consequence of a lower cell surface expression of the Envs, the levels of which were similar in both groups (Fig. 2A), but were linked to a lower ability to bind to CD4 in a cell-to-cell HIV-1 transfer assay (Fig. 3E). We observed that the Envs from LTNP-EC viruses showed a low ability to bind to the cell-surface-expressed CD4 in primary cells and that the extent of binding correlated to their fusion capacity (*P* = 0.0171, Spearman’s test) (Fig. 4B). Considering the chronology of CD4 binding and membrane fusion during HIV-1 entry, this significant correlation provides a causal link between both observations. Therefore, in this LTNP-EC cluster, the reduced Env-mediated fusion efficiency is related to the low ability to interact with the cell-surface-expressed CD4.

The invasion and infection of CD4^+^ T lymphocytes by HIV-1 is a complex process involving many cellular events triggered after the first Env-CD4 interaction that have been the subject of many studies (29, 30, 33, 41). The accumulated evidence indicates that the actin mobilization that occurs before the formation of the fusion pore plays a central role in this process (29, 30, 32). In fact, the actin cytoskeleton is deeply involved in the capping of cell surface receptors for viral infection, which facilitates the interaction with the Env complex and the subsequent fusion pore formation, entry, and infection. These HIV-1 Env-gp120/CD4-mediated actin and receptor reorganization and capping events have been shown to correlate with the infectivity of the virus (29, 30, 32). Moreover, the Env-mediated membrane fusion activity relies directly on the stabilization of a cortex of acetylated α-tubulin, triggered by the first Env-CD4 interaction (33, 34). These two key Env-CD4 signals for HIV-1 fusion, entry, and infection have been a central issue in the present study, where we observed that primary Envs from viruses of the LTNP-EC cluster individuals present nonefficient Envs for the promotion of both cortical F-actin reorganization and capping, with neither signal stabilizing acetylated α-tubulin. These data correlated well with the fact that these Envs are not able to bind CD4 with high affinity. Altogether these data suggest that the low fusion, infection, and replication activities of viruses from this LTNP-EC cluster are due to the inability of the viral Envs to bind to CD4 and to subsequently signal to appropriately rearrange the cortical cytoskeleton to fuse, enter, and infect target cells.

This study supports the role of viral characteristics in the clinical outcome of LTNP-EC individuals. We observed that the genotypic and phenotypic characteristics of the Env proteins from HIV-1 viruses of the cluster of LTNP-ECs were inherited by all the patients’ viruses, which all displayed a low replication capacity and produced, in individuals with different host genotypes (Table 1), the same clinical LTNP-EC phenotype. It is reasonable to assume that because of their poor replicative capacity, these viruses established a low-level primary infection. The low replicative capacity could also affect the transmissibility of these viruses; however, the intravenous route of transmission reported in this cluster could have contributed to overcoming this barrier (Table 1).

The impaired Env functionality favored early immune control of the virus by a strong immunologic response during not only acute but also chronic HIV-1 infection, determining the long-term outcome (18, 20). The Envs from the cluster viruses showed low binding to CD4 and, in parallel, a low signaling activity to modify and reorganize tubulin and actin cytoskeletons, leading to a low fusion activity. These viral characteristics outline for the first time a mechanism that directly involves viral Env-associated functions as being responsible for inefficient performance of the initial steps of the HIV-1 viral cycle, thereby explaining the poor transmissibility and pathogenicity of the viruses from the LTNP-EC cluster under investigation. Our results together with those from a previous work that reported inefficient infection of viruses from LTNP-ECs (11) could indicate that the ability of HIV-1 to establish efficient infection determines the *in vivo* progression of the infection, and as we specifically described here, inefficient Env-mediated functions appear to be responsible for the LTNP-EC phenotype in this cluster of HIV-1^+^ individuals.

Until now, the heritability of HIV-1 virulence has been deduced from transmission pairs or from phylogenetic inferences in large cohorts of viruses in different countries and risk group patients, with widely varying results ranging from 7 to 53% of inheritance (16). In this work, we show that the characteristics of the cluster viral Envs have been inherited by all the viruses from a phylogenetic cluster, even if these patients showed different host markers, as shown by the HLAs (Table 1). We also demonstrated that the viral characteristics influence the clinical phenotype of the infected patients. The heritability of the inefficient functionality of the viral *env* was observed for the first time in viruses from a cluster of patients with the same clinical characteristics and resulted in the same specific nonevolving clinical phenotype. The virus from patient AS7, whose clinical outcome is unknown, shared 11 mutations with the other cluster viruses (35), but it presented three extra mutations that we showed were responsible for the replication capacity of this virus (Fig. 6A and B). These inefficient viral *env* genes of the cluster viruses could be very interesting reagents with which to study new approaches in vaccination programs.

In summary, we have demonstrated for the first time that the low CD4 binding and signaling characteristics of HIV-1 Envs explain the low replication capacity and transmissibility of these viruses. These viral characteristics, particularly in the *env* genes, could be the basis for the lack of clinical progression and the LTNP-EC clinical phenotype in the cluster of HIV-1^+^ individuals studied in this work. These results demonstrate not only the important role of HIV-1 viral properties in the clinical characteristics of the patients, but also, because they were inherited by all viruses in the LTNP-EC cluster, the heritability of viral properties.

## MATERIALS AND METHODS

### Samples studied, *env* nucleotide sequencing, and phylogenetic analysis.

Briefly, 184 *env* nucleotide sequences were analyzed, of which 114 were from samples taken before 1995 from Spanish and North American individuals. The remaining sample sequences (*n =* 70) were obtained from Spanish LTNPs and other viremic patients infected after 2000 (35). Within these samples, we identified a very tight and close cluster of viruses, which was not the result of contamination because the nucleotide sequences were obtained with extreme physical separation measures, in different samples of the patients, and even in different laboratories (35). The analysis and phylogenetic reconstruction of the tree where the viral cluster was identified were previously described (35). Five of the cluster subjects were identified as LTNP-ECs; they were intravenous drug user (IDUs) in the early 1980s, and all lived in Madrid and had a first HIV-1^+^ serology result between 1985 and 1990. The follow-up of patients LTNP_RF_15 and LTNP_RF_21 was not performed in our laboratory, and the only clone studied from patient LTNP_RF_21 was kindly provided by R. Delgado (Hospital Doce de Octubre, Madrid, Spain). Epidemiological and clinical characteristics of patient’s samples are shown in Table 1.

### Ethics statement.

Samples were obtained from participants in previous studies who gave informed consent for genetic analysis studies, and they were registered as a sample collection in the Spanish National Registry of Biobanks for Biomedical Research under no. C.0004030. Consents were approved by the Ethical and Investigation Committees of the Centro Sanitario Sandoval (Madrid) and Hospital 12 de Octubre (Madrid), and the samples were encoded and deidentified in these centers. All clinical investigations were conducted according to the principles expressed in the Declaration of Helsinki. The studies were approved by the Comité de Ética de la Investigación y de Bienestar Animal of the Instituto de Salud Carlos III under no. CEI PI 05_2010-v3 and CEI PI 09-2013.

### Generation of *env* gene expression plasmids.

Several expression plasmids were generated from *env* genes of 4 cluster individuals (LTNP_1, LTNP_3, LTNP_RF_21, and AS7), 4 HIV-1 chronic progressors (I_10, IV_10, I_14, and RIS_06), and 3 laboratory-adapted viruses (SF162, NL4.3, and 89ES_061). The R5-tropic BaL.01-*env* (catalog no. 11445) glycoprotein plasmid was from the NIH AIDS Research and Reference Reagent Program. The *env* genes were amplified by nested PCR from proviral DNA (35). The products were cloned into the pcDNA3.1D/V5-His Topo expression vector (Invitrogen). In total, 9 clones derived from the 4 cluster patients (LTNP_1 clones 8, 10, 12, and 37; LTNP_3 clones 20, 22 and 3; LTNP_RF clone 21; and AS7), 6 clones from chronic progressors (I_10 clones 27 and 28, IV_10 clones 5 and 16, RIS_06 clone 2, and patient I_14 clone 9), and 4 clones from reference samples (SF162, 89ES_061, NL4.3, and BaL.01) were constructed. pcDNA3 plasmids coding for NL4.3-derived mutants D368R and 41.2, which abrogate CD4 binding and gp41-mediated membrane fusion, respectively, were generated by site-directed mutagenesis or by subcloning from original plasmids, as previously described (24). Expression plasmids were transformed in DH5α cells, and clones were sequenced to check the correct insertion of the *env* gene.

### Cells.

The human CEM.NKR-CCR5 permissive cell line (catalog no. 4376), the HEK-293T line (catalog no. 103), and the TZM-bl line (catalog no. 8129) were from the NIH AIDS Research and Reference Reagent Program. Cells were cultured and used as previously described (29, 30, 33, 41).

### Functional analysis of cloned Envs.

The Env expression plasmids were used to transfect 293T cells with the CalPhos mammalian transfection kit (Clontech, Palo Alto, CA) in combination with either a Tat expression plasmid, pTat, for Env expression and fusion assays or with the *env*-defective HIV-1 backbone PSG3 plasmid for viral transfer assays (10). Env expression, viral transfer, and fusion capacities were assessed 24 h after cotransfection by harvesting transfected cells with Versene and washing cell suspensions twice in phosphate-buffered saline (PBS).

### Analysis of cell-surface Env expression.

293T cells were cotransfected with the *env* expression plasmids and the *tat* expression plasmid; 10^5^ cells were incubated with the anti-gp120 antibody 2G12 (4 μg/ml; Polymun, Vienna, Austria) for 40 min at 37°C, washed in PBS, and further incubated with phycoerythrin (PE)-labeled goat anti-human IgG (Jackson ImmunoResearch Laboratories, West Grove, PA) at room temperature for 15 min. Cells were washed with PBS and acquired in a fluorescence-activated cell sorter (FACS) LSRII flow cytometer. Data were analyzed using FlowJo software (Tree Star, Inc., San Carlos, CA). The percentage of Env-expressing cells was used to assess Env expression (10).

### Fusion activity.

To test the fusion capacity of the Env proteins, 293T cells were cotransfected with the *env* expression plasmids and the control *tat* plasmid. A total of 10^4^ cells were mixed at a 1:1 ratio in 96-well plates with CD4^+^ CXCR4^+^ CCR5^+^ TZM-bl cells for 6 h. 293T cells transfected only with *tat* plasmid were used as control. Luciferase activity was measured in a Fluoroskan Ascent (Thermo Fisher Scientific Oy, Vantaa, Finland) using the Brite-Lite luciferase luminescence assay (PerkinElmer, Akron, OH). The percentage of Env-expressing cells and the mean fluorescence intensity (MFI) of these cells were used to calculate relative fluorescence intensity (RFI) as a measure of the number of cell-surface Env molecules as described previously (46): RFI = % of Env^+^ cells × MFI of Env^+^ cells The ratio of total fusion to RFI was considered the fusogenicity index (FI), which estimates the fusogenic capacity of an individual Env irrespective of the level of expression (10).

### Viral transfer activity.

To test viral transfer activity, which exclusively depends on the binding of gp120 to the CD4 molecule, *env* expression plasmids were cotransfected with the defective pSG3 plasmid in 293T cells. A total of 10^5^ cells were mixed at a 1:1 ratio in 96-well plates with primary CD4^+^ T cells freshly isolated from donors by negative selection (CD4^+^ T cell isolation kit II, human; Miltenyi Biotec, Inc., Bergisch Gladbach, Germany). Viral transfer was assessed after 2 h of incubation, staining cells with the anti-HIV-1 p24 antibody Kc57 (Coulter clone; Beckman Coulter, Inc., Brea, CA) (42). Cells were acquired in a FACS LSRII flow cytometer, and the content of p24 in gated CD4^+^ T cells and gated 293T cells was analyzed using FlowJo software (Tree Star, Inc.) and expressed as the percentage of p24-positive cells. The percentage of p24^+^ 293T cells was used as a control of transfection efficiency, and the percentages were similar among experiments. The frequency of p24^+^ CD4^+^ T cells was a direct measure of the amount of the viruses bound to and taken up by these cells.

### Production of viral particles with luciferase reporter pseudoviruses.

Replication-deficient luciferase HIV-1 viral particles (luciferase reporter pseudoviruses) were obtained as described previously (29, 41, 47), using the luciferase-expressing reporter virus HIV/Δ*nef*/Δ*env*/*luc*^+^ (pNL4-3.Luc.R-E provirus bearing the luciferase gene inserted into the *nef* open reading frame [ORF] and which does not express *env*; catalog no. 6070013, NIH AIDS Reagent Program) and the described *env* expression plasmids from the different HIV-1 individuals or the reference *env* plasmids studied. As a control for non-CD4 entry and infection and viral production, when indicated, cotransduction of the pNL4-3.Luc.R-E provirus (20 μg) vector with the pHEF-VSV-G vector (10 μg; NIH AIDS Reagent Program) was used to generate nonreplicative viral particles that fuse with cells in a vesicular stomatitis virus glycoprotein (VSV-G)-dependent manner (41). Viral stocks were normalized by p24-Gag content measured by enzyme-linked immunosorbent assay (ELISA [Innotest HIV antigen MAb, reference no. 80563; Fujirebio Europe, Göteborg, Sweden]).

### Luciferase viral entry and infection assay.

CEM.NKR-CCR5 cells (9 × 10^5^ cells in 24-well plates with 20 μg/ml Polybrene) were infected with 500 ng of p24 of luciferase reporter pseudoviruses in 1 ml total volume with RPMI 1640 for 2 h (by centrifugation at 1,200 × *g* at 25°C) and subsequent incubation for 4 h at 37°C, as previously described (29, 41, 47). Unbound virus was then removed by washing the infected cells. After 48 h of infection, luciferase activity was measured using a luciferase assay kit (Biotium, Hayward, CA) with a microplate reader (Victor X5; PerkinElmer, Waltham, MA). When indicated, permissive cells were pretreated with an anti-CD4 neutralizing MAb (5 μg/ml; EBioscience, San Diego, CA).

### *In vitro* mutagenesis.

Mutant AS7 clones (AS7_I140T, AS7_V279A, and AS7_I400T) were obtained by site-directed mutagenesis using the QuikChange site-directed mutagenesis kit (Stratagene) by following the manufacturer’s instructions. Mutations were verified by DNA sequencing of the ENV-coding region.

### Signaling activity assays.

(i) Western blot analysis of HIV-1 Env-mediated α-tubulin acetylation was studied in CEM.NKR-CCR5 cells (1 × 10^6^ cells) incubated with 500 ng of p24 of luciferase reporter pseudoviruses for 1 h at 37°C (33). Membranes were probed with the anti-α-tubulin B-5-1-2 monoclonal antibody (MAb) and the anti-acetylated α-tubulin 6-11B-1 MAb (both from Sigma-Aldrich, St. Louis, MO) and secondary antibodies conjugated with horseradish peroxidase (HRP) (Dako, Glostrup, Denmark). The increase in α-tubulin acetylation was quantified and expressed as the ratio of the intensities of the acetylated α-tubulin to the total α-tubulin bands, as described previously (33, 48). (ii) Immunofluorescence of HIV-1 Env-mediated acetylated α-tubulin and the F-actin capping assay was analyzed in CEM.NKR-CCR5 cells (1 × 10^6^ cells/point) incubated with HIV-1 luciferase reporter pseudoviruses (29, 30, 33), as described for Western blot analysis. Coverslips were mounted in Mowiol antifade (Dako) and imaged in ***xy*** midsections in a FluoView FV1000 confocal microscope through a 1.35 NA objective (60×) (Olympus, Center Valley, PA) for high-resolution imaging of fixed treated cells. The final images and molecule codistributions were analyzed and quantified with MetaMorph software (Universal Imaging, Downington, PA).

### Statistical analysis.

Data and statistical analyses were performed using GraphPad Prism, version 6.07 (GraphPad Software, Inc.). Significance when comparing 2 groups was determined with a 2-tailed nonparametric Mann-Whitney *U* test or by nonparametric Dunn’s test for multiple comparisons. Student’s *t* test was used for the comparison of the signaling activity. A nonparametric Spearman’s test was used to calculate correlations.

### Data availability.

Data presented have been generated by the three laboratories. Any researcher will be able to access primary data upon request to jblanco@irsicaixa.es, avalenzu@ull.edu.es, or clopez@isciii.es.
